# Discovery of new *Schistosoma mansoni* aspartyl protease inhibitors by structure-based virtual screening

**DOI:** 10.1590/0074-02760230031

**Published:** 2023-09-01

**Authors:** Bárbara Figueira Gomes, Mario Roberto Senger, José Teófilo Moreira-Filho, Fabio Jorge de Vasconcellos, Rafael Ferreira Dantas, Raymond Owens, Carolina Horta Andrade, Bruno Junior Neves, Floriano Paes Silva-Junior

**Affiliations:** 1 Fundação Oswaldo Cruz-Fiocruz, Instituto Oswaldo Cruz, Laboratório de Bioquímica Experimental e Computacional de Fármacos, Rio de Janeiro, RJ, Brasil; 2 Universidade Federal de Goiás, Faculdade de Farmácia, Laboratório de Planejamento de Fármacos e Modelagem Molecular, Goiânia, GO, Brasil; 3 University of Oxford and Rosalind Franklin Institute, Oxfordshire, UK; 4 Universidade Federal de Goiás, Faculdade de Farmácia, Laboratório de Quimioinformática, Goiânia, GO, Brasil

**Keywords:** Schistosoma mansoni, drug discovery, hit identification, aspartic protease, virtual screening

## Abstract

**BACKGROUND:**

Schistosomiasis is a neglected tropical disease caused by trematodes of the genus *Schistosoma*, with a limited treatment, mainly based on the use of praziquantel (PZQ). Currently, several aspartic proteases genes have already been identified within the genome of *Schistosoma* species. At least one enzyme encoded from this gene family (*Sm*AP), named *Sm*CD1, has been validated for the development of schistosomicidal drugs, since it has a key role in haemoglobin digestion by worms.

**OBJECTIVE:**

In this work, we integrated a structure-based virtual screening campaign, enzymatic assays and adult worms *ex vivo* experiments aiming to discover the first classes of *Sm*CD1 inhibitors.

**METHODS:**

Initially, the 3D-structures of *Sm*CD1, *Sm*CD2 and *Sm*CD3 were generated using homology modelling approach. Using these models, we prioritised 50 compounds from 20,000 compounds from ChemBridge database for further testing in adult worm aqueous extract (AWAE) and recombinant *Sm*CD1 using enzymatic assays.

**FINDINGS:**

Seven compounds were confirmed as hits and among them, two compounds representing new chemical scaffolds, named **5** and **19**, had IC_50_ values against *Sm*CD1 close to 100 μM while presenting binding efficiency indexes comparable to or even higher than pepstatin, a classical tight-binding peptide inhibitor of aspartyl proteases. Upon activity comparison against mammalian enzymes, compound **50** was selective and the most potent against the AWAE aspartic protease activity (IC_50_ = 77.7 μM). Combination of computational and experimental results indicate that compound **50** is a selective inhibitor of *Sm*CD2. Compounds **5**, **19** and **50** tested at low concentrations (10 uM) were neither cytotoxic against WSS-1 cells (48 h) nor could kill adult worms *ex-vivo*, although compounds **5** and **50** presented a slight decrease on female worms motility on late incubations times (48 or 72 h).

**MAIN CONCLUSION:**

Overall, the inhibitors identified in this work represent promising hits for further hit-to-lead optimisation.

Schistosomiasis is a parasitic disease caused by trematodes of the genus *Schistosoma*, which has several species, including *S. mansoni*. More than 236.6 million people needed treatment for schistosomiasis in 2019, according to the World Health Organization (WHO).^1^ Praziquantel (PZQ) has been the drug of choice for the treatment of schistosomiasis for more than 40 years.^2^ Park et al.^3^ demonstrated that PZQ activates a transient ion channel receptor called TRPM_PZQ_. This receptor promotes calcium influx and parasite paralysis. Despite a known mode of action, PZQ does not act on immature forms (eggs, larvae and juvenile worms) of *S. mansoni*.^4^ ^,^ ^5^ Moreover, the bitter taste and size of PZQ tablets are also limitations, mainly for children’s treatment. Finally, it has already been observed that the use of large-scale chemotherapy through successive treatments may result in the emergence of resistant isolates, through selective drug pressure.^6^ ^,^ ^7^ Thus, the discovery of new drugs with new mechanisms of action would be of great value to the therapeutic arsenal against this important neglected tropical disease.

The development of the larval form (schistosomula) up to adulthood, as well as nutrition, reproduction, and growth of worms, requires proteolytic enzymes, which digest the proteins obtained from the host.^8^ ^,^ ^9^ ^,^ ^10^ Parasites such as *Schistosoma* spp. produce a cathepsin D-like aspartyl protease 1 (*Sm*CD1)^11^ that, along with cysteinyl proteases, have a pivotal role in the early stages of the *S. mansoni* digestion cascade.^10^ ^,^ ^12^ Studies from our group showed that *S. mansoni* presents a family of aspartyl proteases genes (collectively called *Sm*APs).^13^ Although only one of these genes, named *Sm*CD1, has been formally described in the literature,^11^ ^,^ ^12^ ^,^ ^14^ two new *Sm*CDs were identified and named *Sm*CD2 and *Sm*CD3.^13^ Araujo-Montoya et al.^14^ developed an expression system in HEK 293T mammalian cells and performed a biochemical and biophysical characterisation of the recombinant, r*Sm*CD1 enzyme. The purified enzyme showed activity in degrading human haemoglobin and cleaving a commercial aspartyl protease peptide substrate, as well being inhibited by pepstatin, a classic inhibitor of aspartyl proteases.

Previously, it has been observed that the RNAi-mediated reduction in transcriptional levels of *Sm*CD1 leads to phenotypic changes in the parasite, including significant *in vitro* growth retardation.^11^ ^,^ ^15^ In addition, the dark pigment of hemozoin, which is the product of haemoglobin proteolysis, did not accumulate in the gastrodermis of the treated parasites. Thus, these studies indicate that *Sm*CD1 is an essential enzyme in the stages of schistosomes parasitising the mammalian host. As such, *Sm*CD1 can be considered a validated and exceptionally promising target for the development of drugs against schistosomiasis. Until this moment, known inhibitors of *Sm*CD1 are scarce. Only one HIV-1 protease inhibitor and few statin-based peptides are known to inhibit this enzyme or it’s orthologue in *S. japonicum*.^13^ ^,^ ^15^ ^,^ ^16^ ^,^ ^17^

The technological advances in the computational field have contributed to increase the efficiency of the drug discovery process.^18^ ^,^ ^19^ In this context, along with advances in structural biology, the virtual screening (VS) technique emerged as a strategy for the identification of new bioactive substances that is widely used in the early stages of the drug discovery pipeline.^20^ The main reason behind the success of this computational approach is the possibility of prioritising compounds from a large virtual library based on their predicted biological activity. Thus, there is a significant decrease in the number of experimental trials to be performed and consequently in the total cost and time of the whole process.^21^ Several active compounds against *S. mansoni* have already been identified by VS, including protease inhibitors.^19^ ^,^ ^22^ ^,^ ^23^

In this work, we performed a structure-based VS campaign to identify the first classes of small molecule inhibitors of *SmCD1*. To achieve this goal, we pursued the following steps: (i) to build the 3D-structures of *Sm*CD1, *Sm*CD2 and *Sm*CD3 using homology modelling; (ii) to perform a docking-based VS with 20,000 compounds available on MicroFormat and DIVERSet-EXP datasets; (iii) to perform experimental validation of the best scored computational hits against the adult worm aqueous extract (AWAE) and recombinant *Sm*CD1; (iv) to assess inhibitor selectivity by comparing to inhibition of mammalian homologous enzymes; and (v) to evaluate the *in vitro* effect of hit compounds on *ex vivo* adult *S. mansoni* worms and cultured WSS-1 cells.

## MATERIALS AND METHODS

Computational

*Homology modelling* - The amino acid sequences of *Sm*CD1 (ID: P91802), *Sm*CD2 (ID: P91802), and *Sm*CD3 (ID: P91802) were retrieved from the UniProt database^24^ ^,^ ^25^ and used as targets for homology modelling. Templates for modeling were selected from the Protein Data Bank (PDB)^26^ using as criteria the highest sequence identity with the targets, best resolution of the available experimental structure and biological function relevance. Initially, a target-template alignment was calculated by T-Coffee server.^27^ Subsequently, 50 homology models were built for each protein using “satisfaction of spatial restrains” method implemented in MODELLER v.8.^28^ In this approach, a 3D model is obtained by satisfying main-chain and side-chain dihedral angles of all non-hydrogen atoms derived from the alignment and expressed as probability density functions (PDFs). The 3D models were obtained by optimisation of the molecular PDF such that the model violates the input restraints as little as possible.^28^ Finally, the generated models were exported to SAVES server (http://services.mbi.ucla.edu/SAVES/) and their overall stereochemical and structural quality were checked according to PROCHECK,^29^ ^,^ ^30^ VERIFY,^31^ ^,^ ^32^ and PROVE^33^ scores.

*Ligand preparation* - The 3D structures of the small molecules were imported to MarvinSketch v.6.3.1 (ChemAxon, Budapest, Hungary, http://www.chemaxon.com) and protonated at pH = 3.5 ± 0.5. Subsequently, up to 1,000 conformers were generated for each structure using OMEGA v.3.0.0.,^34^ ^,^ ^35^ and the AM1-BCC charges^36^ were added using QUACPAC v. 1.7.0.2.^37^

*Protein preparation* - The Protein Preparation Wizard program, which is available on Maestro workspace v.9.3 (Schrödinger, LCC), was employed to process the 3D structures of the *Sm*CDs. This procedure involves the addition of hydrogen atoms to the proteins and the adjustment of bond orders and formal charges. Additionally, the protonation states of polar amino acid residues were forecasted at pH = 3.5 ± 0.5 using PROPKA v.3.1. In contrast, Asp219 and Asp33’s protonation states, which are part of the catalytic triad, were manually adjusted to their neutral form based on experimental findings for other aspartic proteases within the same family^38^ ^,^ ^39^ ^,^ ^40^ ^,^ ^41^ and following the catalytic mechanism proposed by Veenrpandian et al.^42^

*Molecular docking* - The 3D structures of *Sm*CD1, *Sm*CD2, and *Sm*CD3 were subjected to the grid-generation in OEDocking suite v.3.2.0.2^43^ ^,^ ^44^ ^,^ ^45^ by fixing a box on the geometrical center of catalytic triads (Asp33, Thr34, and Gly35). Details of grid boxes can be found in Supplementary data (Table I). Finally, molecular docking calculations were performed in FRED program^43^ ^,^ ^44^ ^,^ ^45^ using standard and high-resolution protocols as well as the ChemGauss4 score function.^44^ ^,^ ^45^ ^,^ ^46^ High resolution protocol improves every possible rotation and translation of each conformer of the ligand being docked within a box enclosing the active site.^43^ ^,^ ^44^ ^,^ ^45^ During the docking, any pose examined by the exhaustive search that does not fit within the outer shape (~ 5 Å of the triad) of the corresponding grid box was rejected.

*Virtual screening (VS)* - After defining the molecular docking protocols for each target, they were employed as filters for the virtual screening of 20,000 compounds available in the MicroFormat and DIVERSet-EXP collections of the ChemBridge database (http://www.chembridge.com/). Following that, the KNIME workspace v.1.3.5 was utilised to import the top 2.5% virtual hits, which were then assessed for their metabolic stability against nine cytochrome P450 enzymes (CYP3A4, CYP2D6, CYP2C9, CYP2C19, and CYP1A2) using our in-house machine learning models. Meanwhile, the hERG blockage potential was predicted using the Pred-hERG web server.^47^ ^,^ ^48^ To develop the cytochrome P450 machine learning models, we used the Random Forest algorithm and ECFP4 fingerprints, which were implemented in KNIME v.1.3.5. Then, a medicinal chemistry inspection filter based on using Tanimoto coefficient and MACCS keys was employed to ensure the structural novelty of virtual hits in relation to known anti-schistosomal compounds available on ChEMBL database (ID: CHEMBL612893). At the end of this process, the docking poses were visually inspected, and the best virtual hits were selected based on interactions with the catalytic triad.

Experimental

*Chemicals* - The reagents bovine cathepsin D (*BtCD*) (C3138) and pepstatin A (P5318) were obtained from Sigma-Aldrich, porcine pepsin (0151-17 / 215110) from Difco, Abz-AIAF/FSRQ-EDDnp fluorescent substrate (102023-1) from GenOne and E-64 (78434) from Thermo Scientific. The other reagents used, as salts, buffers, 3-[(3-cholamidopropyl)dimethylammonio]-1-propanesulfonate (CHAPS), dimethyl sulfoxide (DMSO), culture medium were of the PA grade or the highest level of purity available on the market.

*Parasites* - Swiss Webster mice were used to obtain *S. mansoni* adult worms. The animal infection was described previously by our group.^49^ All experiments were carried out in accordance with the Oswaldo Cruz Foundation Institutional Ethics Committee for Laboratory Animals (CEUA / FIOCRUZ, Brazil, L-044/15).

*Preparation of adult worm aqueous extract (AWAE)* - The extract was prepared from the homogenisation of 1 mL of worms, containing both male and female, in 5 mL of phosphate buffered saline (PBS) + 1% CHAPS, at pH 7.4, using a Turrax disperser. This volume of parasites is roughly equivalent to 20 adult parasites. The homogenate was centrifuged at 20,000 g for 1 hour at 4ºC, and the supernatant stored at -80ºC. An aliquot was used for the protein dosing by the Lowry method (DC^TM^ protein assay - BIO-RAD).

*SmCD1 cloning, expression and purification* - Expression of the recombinant enzyme was previously performed by our group^14^ by transfection of the vector with recombinant pro*Sm*CD1 gene (pro*Sm*CD1r) into culture bottles containing human embryonic kidney (HEK293) cells. After an incubation period of three to five days, the culture medium of the HEK293 cells was collected, filtered, and used for the purification of the recombinant protein. A HisTrap FF column in-tandem with a HiLoad 16/60 Superdex column, connected to an Äkta Xpress system were used for the purification of the protein.

*SmCD1 enzymatic assay* - Initially, 2.5 μg of *Sm*CD1 was added to a reaction medium containing 50 mM sodium acetate buffer with 50 mM NaCl at pH 3.5. Next, this medium was incubated with either test compounds (10 µM), pepstatin (1 - 500 µM), as a positive control, or DMSO (0,1%), as negative control, for 10 minutes, at 37ºC. After incubation, to start the reaction, 10 μM of the substrate octapeptide fluorescent (FRET) Abz-AIAF/FSRQ-EDDnp (102023-1, GenOne) was added to the medium. The substrate hydrolysis reaction product was continuously detected (λex = 310 nm and λem = 420 nm), using a Flex Station III microplate reader (Molecular Devices), during the initial 10 min of the reaction.

All enzymatic assays in this work were performed as described above, and repeated at least three times, each condition in triplicate. In tests involving screening of compounds, the percentage of inhibition of enzymatic activity was calculated considering the mean of the negative control as 100% activity.

*AWAE aspartic protease activity assay* - For assaying the aspartyl protease activity present in the AWAE, the same protocol described above for *Sm*CD1 was followed, except for 20 µg of total proteins present in AWAE were added instead of the recombinant *Sm*CD1 enzyme. Additionally, 5 µM of E-64, an inhibitor of cysteinyl proteases, was added to prevent enzymes of this class present in the AWAE^15^ to also contribute to substrate hydrolysis on the acidic pH of the assay.

*Specificity assays toward mammalian proteases* - For recombinant *Bt*CD and porcine pepsin, the same protocol described above for *Sm*CD1 was followed, except for 10 μg of *Bt*CD or 5 μg of pepsin were added.

*Determination of IC*_*50*_ *values* - For IC_50_ (inhibitor concentration in which the enzymatic activity is reduced by 50%) determination assays, serial dilutions of the inhibitors were performed, ranging from 1 µM to 500 µM. The IC50 values were determined by fitting *Sm*CD1 activity data into the 4-parameter logistic equation.

*Cytotoxicity assays on human WSS-1 cells* - The cytotoxicity of compounds 5, 19 and 50 was evaluated against normal epithelial cells derived from human kidney (WSS-1, ATCC™ CRL-2029). Cells were seeded in 96-well plates, at 5x10^4^ cells/mL, in DMEM high glucose medium supplemented with 10% foetal bovine serum (FBS). After adherence period of 24 h, at 37ºC and 5% CO_2_, the cells were treated with compounds at 10 µM or 100 μM concentrations and incubated for 44 h. After this time, resazurin solution was added to cells culture, at a final concentration of 0.01 mg/mL. The cells returned to the incubator for another 4 h to complete 48 h total incubation time.^50^

Fluorescence readings of resorufin (λex = 560 nm, λem = 590 nm) were taken immediately (F0) and 4 hours (F4) after resazurin addition, using Flex Station III. The percentage of viable cells at the end of the experiment, that is, after compounds exposure for 48h, was calculated as follows:

<mml:math><mml:mi>V</mml:mi><mml:mi>i</mml:mi><mml:mi>a</mml:mi><mml:mi>b</mml:mi><mml:mi>l</mml:mi><mml:mi>e</mml:mi><mml:mi> </mml:mi><mml:mi>c</mml:mi><mml:mi>e</mml:mi><mml:mi>l</mml:mi><mml:mi>l</mml:mi><mml:mi>s</mml:mi><mml:mo>=</mml:mo><mml:mfrac><mml:mrow><mml:mi>F</mml:mi><mml:mn>4</mml:mn><mml:mo>-</mml:mo><mml:mi>F</mml:mi><mml:mn>0</mml:mn><mml:mi mathvariant="normal"> </mml:mi><mml:mi mathvariant="normal">t</mml:mi><mml:mi mathvariant="normal">r</mml:mi><mml:mi mathvariant="normal">e</mml:mi><mml:mi mathvariant="normal">a</mml:mi><mml:mi mathvariant="normal">t</mml:mi><mml:mi mathvariant="normal">e</mml:mi><mml:mi mathvariant="normal">d</mml:mi></mml:mrow><mml:mrow><mml:mi>F</mml:mi><mml:mn>4</mml:mn><mml:mo>-</mml:mo><mml:mi>F</mml:mi><mml:mn>0</mml:mn><mml:mi mathvariant="normal"> </mml:mi><mml:mi mathvariant="normal">c</mml:mi><mml:mi mathvariant="normal">o</mml:mi><mml:mi mathvariant="normal">n</mml:mi><mml:mi mathvariant="normal">t</mml:mi><mml:mi mathvariant="normal">r</mml:mi><mml:mi mathvariant="normal">o</mml:mi><mml:mi mathvariant="normal">l</mml:mi></mml:mrow></mml:mfrac><mml:mi mathvariant="normal"> </mml:mi><mml:mi mathvariant="normal">x</mml:mi><mml:mi mathvariant="normal"> </mml:mi><mml:mn>100</mml:mn><mml:mi mathvariant="normal"> </mml:mi></mml:math>

*Schistosoma mansoni adult worms ex vivo assay* - The effect of 10 µM of the test compounds or PZQ (positive control) on adult *S. mansoni* worms motility for up to 72 h of incubation at 37ºC and 5% CO_2_ was evaluated by a high content assay previously developed by our group.^50^ Image analysis was carried out in a customised pipeline of the open-source Cellprofiler software v. 4.2.1^51^ and consisted of quantifying parasites area (Area measurement of MeasureObjectSizeShape module) and its displacement over pairs of consecutive images (false negative rate measurement of MeasureObjectOverlap module). These data were then modeled by a multilevel generalised linear statistical model. The effects of the experimental treatments on parasites motility were estimated from this model by the integrated nested laplace approximation (INLA) method available in INLA package of R (https://www.r-inla.org). The results were expressed as percentual relative motility considering the estimated effect of the negative control group (0.2% DMSO) for each incubation time as 100%.

*Graphics and statistical analysis* - GraphPad Prism version 5.04 (GraphPad Software Inc., San Diego, USA) was used for statistical analysis. The results were expressed as mean ± standard error. Differences were considered significant when p < 0.05.

## RESULTS AND DISCUSSION

**Fig. 1:**
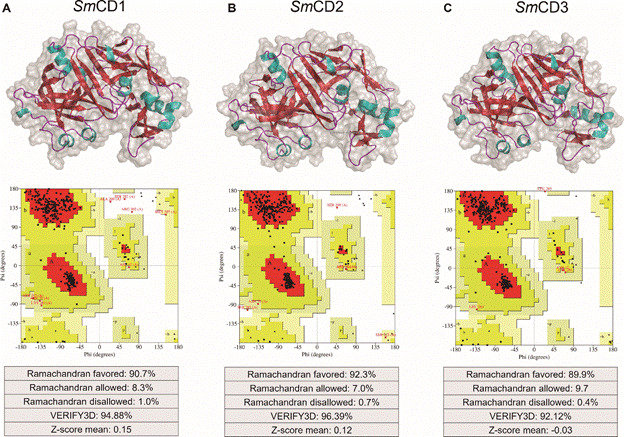
predicted 3D structures of *Schistosoma mansoni* cathepsin D-like aspartyl protease 1 (*Sm*CD1, (A), 2 (*Sm*CD2, (B) and 3 (*Sm*CD3, (C), generated by SWISS-MODEL. Ramachandran plots for each homology model were obtained using PROCHECK and show the dihedral angles Psi and Phi of amino acid residues. Red represents most favoured regions in plot; yellow represents additional allowed regions; beige represents generously allowed regions; and white areas are disallowed regions.

*Homology modelling* - The 3D structures of *Sm*CD1, *Sm*CD2, and *Sm*CD3 were not available on the PDB at the time this work was conducted. Therefore, homology models were built using the crystal structure of human cathepsin D (PDB ID: 1LYB^52^) as template, since it shared identity of 57% and the highly conserved catalytic triad (Asp33, Thr34, and Gly35). Subsequently, the quality of obtained 3D models (Fig. 1) was analysed for various levels of structural information.

Initially, PROCHECK analysis was performed to determine stereochemical quality of models by analysing residue by residue geometry and overall structure geometry.^29^ ^,^ ^30^ Analyses of dihedral angles *phi* against *psi* of amino acid residues (see Ramachandran plots in Fig. 1) indicated that 92.3-89.9% of residues of 3D models are within the most favoured regions, 9.7-7.0% of residues are within the additional allowed regions, only 1.0-0.4% of residues are within the disallowed regions.

Subsequently, PROVE analysis calculated the atom volumes of each *Sm*CD structure using an algorithm that treats the atoms like hard spheres and considered a statistical Z-score deviation in relation to high resolution structures (2.0 Å or better) deposited in PDB.^33^ As shown in Fig. 1, all models had Z-score values close to 0, reflecting the accuracy of homology modelling protocol used in this work.

Further, VERIFY-3D was used to determine the compatibility of 3D models with its own amino acid sequence by assigning a structural class based on its location and environment (alpha, beta, loop, polar, nonpolar, etc.) and comparing the results to good structures.^31^ ^,^ ^32^ As a result, 96.39-92.12% of the residues had average 3D-1D scores ≥ 0.2. This suggests that the 3D models investigated in this work have overall self-consistency in terms of sequence-structure compatibility.

**Fig. 2:**
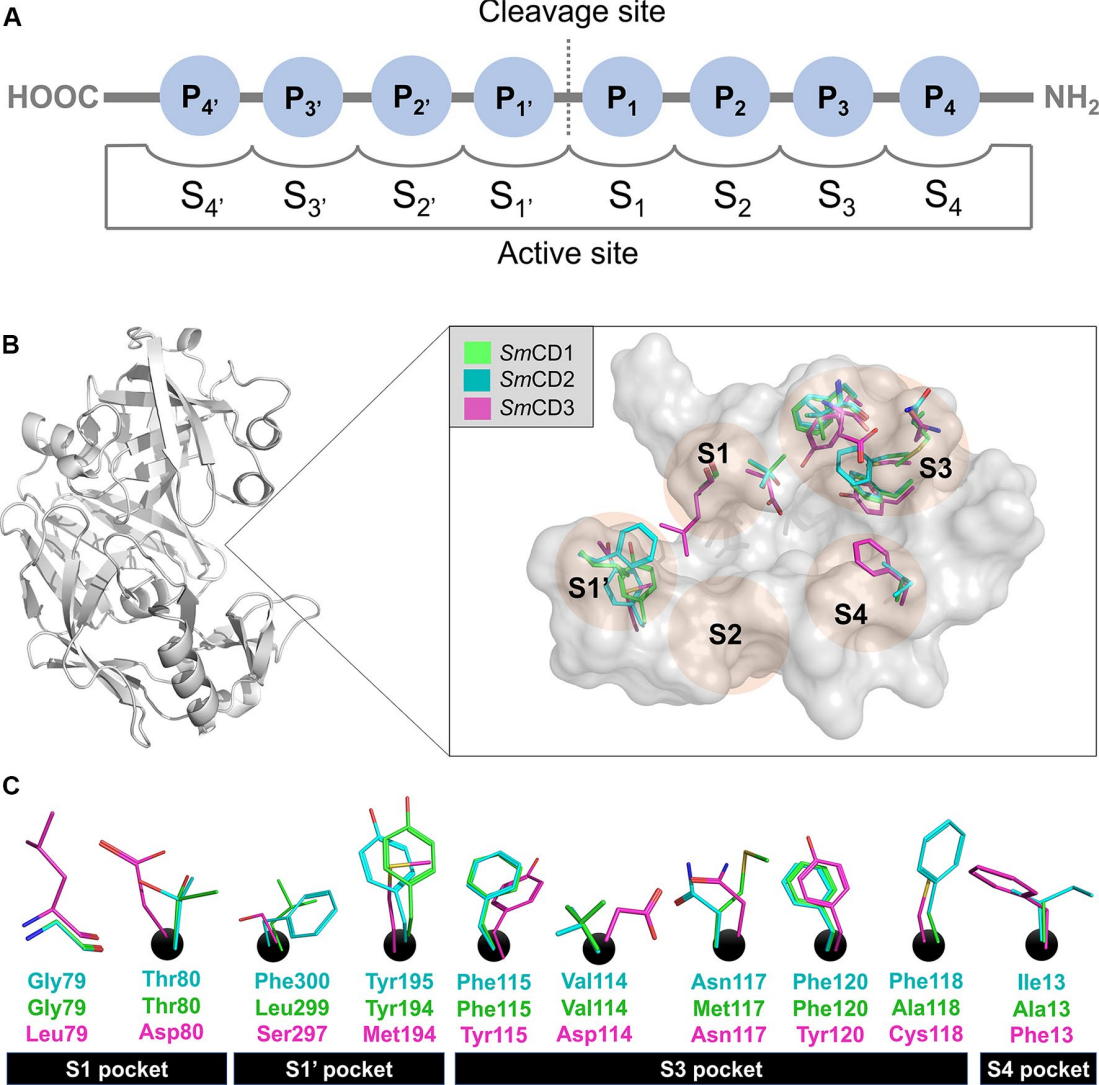
(A) A diagram illustrating the binding of a substrate to the active site of *Schistosoma mansoni* cathepsin D-like aspartyl proteases (*Sm*CDs), wherein the positions are named based on the cleavage site’s location.^16^ (B-C) A representation depicting the primary variations among the active site residues of the enzymes *Sm*CD1, *Sm*CD2, and *Sm*CD3.

As depicted in Fig. 2A, the active site of *Sm*CDs consists of adjoining pockets denoted as -S4’-S3’-S2’-S1’-S1-S2-S3-S4-. These pockets are chemical and spatial complements of the substrate amino acid residues, consecutively numbered as -P_4’_-P_3’_-P_2’_-P_1’_-P_1_-P_2_-P_3_-P_4_-.^16^ ^,^ ^53^ The enzyme catalyses hydrolysis of the substrate scissile bond located between the P_1’_ and P_1_. Usually, aspartic protease inhibitors that bind to the S1-S4 pockets are frequently encountered, while the S1ʹ-S4ʹ pockets typically remain unoccupied.

To explore the feasibility of the suggested binding site, active site similarity profiles were validated by 3D alignment of the *Sm*CDs (Fig. 2B). The alignment of SmCD1 and SmCD2 residues in Fig. 2C highlights a significant degree of preservation of the general topology of S1’-S1-S2-S3-S4 residues. Conversely, the S1 (Leu79 and Asp80), S1’ (Ser297 and Met194), S3 (Tyr115, Asp114, Asn117, Tyr120, and Cys118), and S4 (Phe13) pockets of *Sm*CD3 exhibit marked physicochemical disparities when contrasted with *Sm*CD1 and *Sm*CD2 (Fig. 2C). Based on these dissimilarities, it appears that the active sites of *Sm*CD1 and *Sm*CD2 enzymes are comparatively more solvent-accessible than that of *Sm*CD3.

**Fig. 3:**
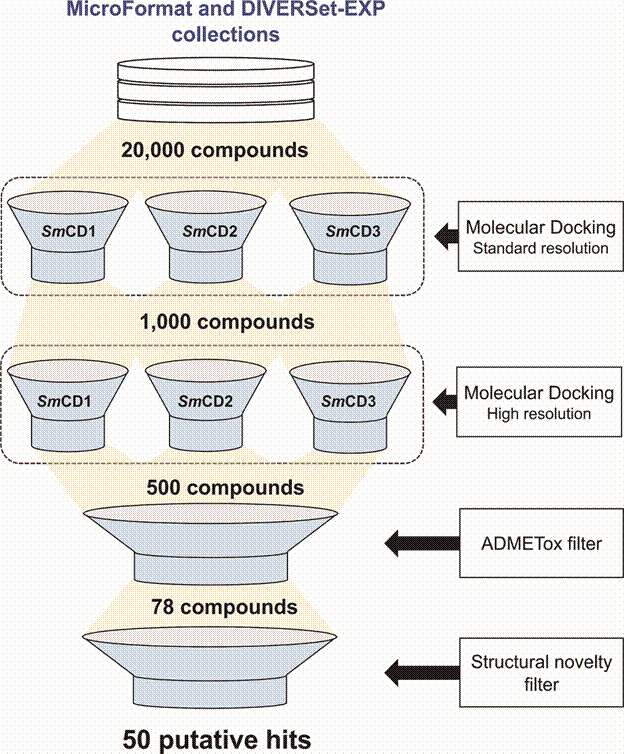
virtual screening workflow used for identifying potential *Schistosoma mansoni* cathepsin D-like aspartyl protease (*Sm*CD) inhibitors.

*Structure-based virtual screening* - The VS of new *Sm*CDs inhibitors was carried out following the workflow presented in Fig. 3.

Initially, 20,000 compounds with lead-like properties available on MicroFormat and DIVERSet-EXP collections of ChemBridge were downloaded and standardised for VS. Then, the standard and high-resolution docking protocols were used as filters to prioritise compounds with potential *Sm*CD1, *Sm*CD2, and/or *Sm*CD3 inhibitory activity. Docking assays against these three enzymes were performed with the rationale that a compound inhibiting multiple *Sm*AP targets should maximise the potential schistosomicidal activity of putative hits. The top 2.5% ranked hits (ChemGauss4 scores ranging between -7.00 and -14.00) were selected for further filtering. The compounds were then evaluated by predicting a panel of pharmacokinetic and toxicity properties including CYP450 metabolic stability and inhibition and hERG blockage.^47^ ^,^ ^48^ At the end of the VS workflow, 50 putative hits representing new chemical scaffolds for schistosomiasis were visually inspected and purchased for experimental validation.

**Fig. 4:**
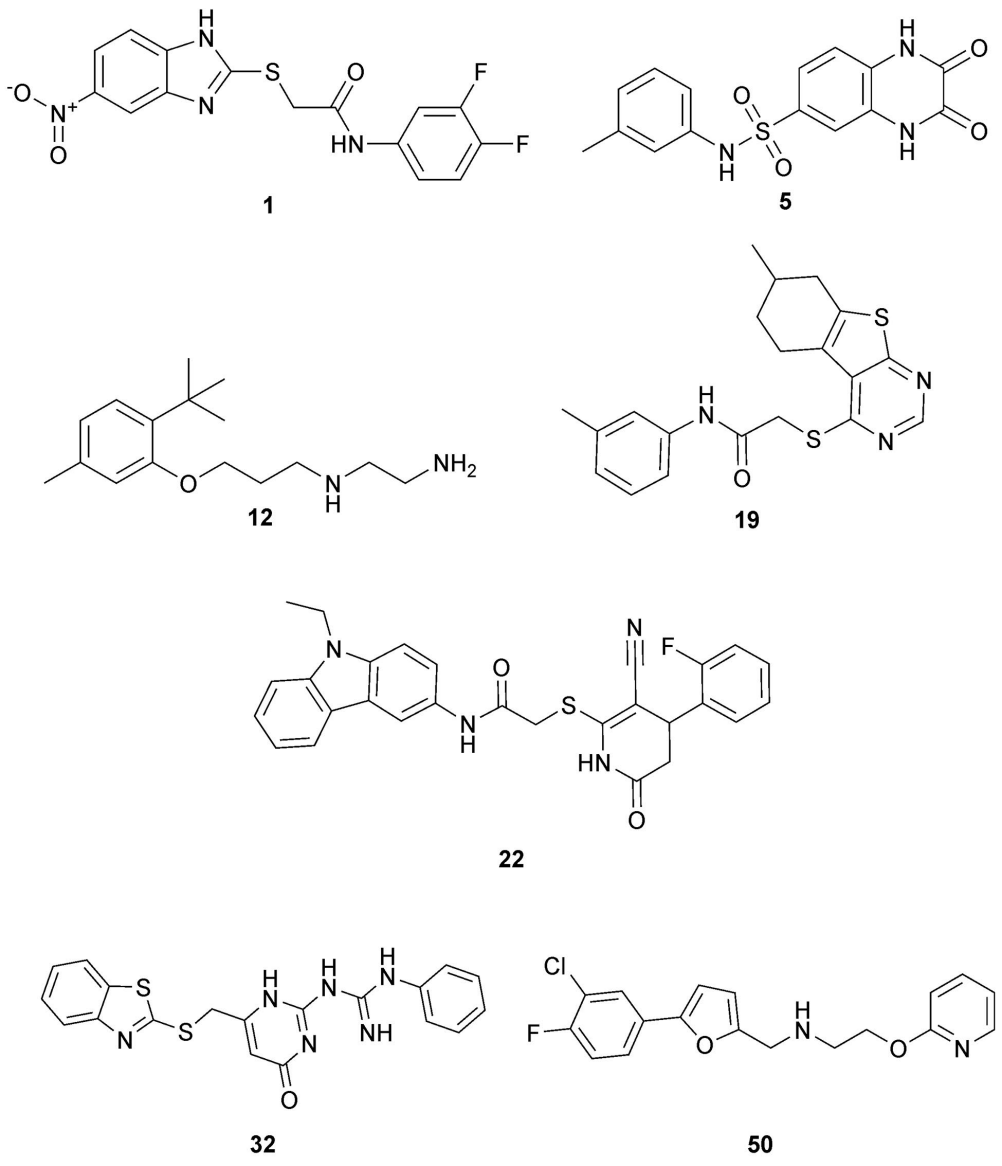
chemical structures of active compounds on aspartic protease inhibititon screening.

*Experimental validation* - The inhibitory activity of the 50 putative hits, at 10 µM, was experimentally investigated in porcine pepsin, *Sm*CD1, and AWAE aspartic protease activity assay, using pepstatin as a positive control [Supplementary data (Table II)]. The AWAE assay captures the combined activities of all *Sm*APs present in schistosome adult worms, while excluding the concurrent cysteinyl protease activity, due the addition of the broad-spectrum E-64 inhibitor. Among the 50 compounds tested, seven compounds (**1**, **5**, **12**, **19**, **22**, **32** and **50,** Fig. 4) were considered active based on the ≥ 25% inhibition of any of the aspartic protease activity [Supplementary data (Table II)].

Following the initial screening, the seven primary hits were selected for determining IC_50_ against pepsin, *Sm*CD1, and AWAE (Table).

**TABLE:** Inhibitory potencies of seven primary hits against the adult worm aqueous extract (AWAE), *Schistosoma mansoni* cathepsin D-like aspartyl protease 1 (*Sm*CD1) and pepsin. Confidence intervals (95% level) are shown in parentheses

Compound	IC_50_ (µM)		
	Pepsin	AWAE	*Sm*CD1
1	> 500	> 500	> 500
5	92.6 (81.6 to 108)	135.1 (^*^ to 240)	83.6 (75.1 to 98.6)
12	231 (215 to 248)	315 (300 to 338)	> 500
19	23.1 (14.8 to 36.6)	> 500	102.5 (77.7 to 138)
22	38.0 (20.7 to 59.4)	> 500	> 500
32	72.5 (35.3 to 309.5)	> 500	> 500
50	> 500	77.7 (63.9 to 104)	> 500

*The lower limit could not be determined.

**Fig. 5:**
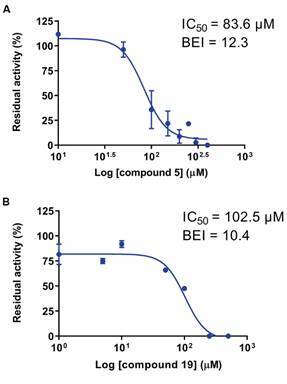
concentration-response curves for *Schistosoma mansoni* cathepsin D-like aspartyl protease 1 (*Sm*CD1) inhibition by compounds **5** (A) and **19** (B). Error bars: mean ± standard deviation (SD) of three independent replicates. Binding efficiency index (BEI) = pIC50/MW(kDa).

Among primary hits, compounds **5** (2,3-dioxo-N-(m-tolyl)-1,2,3,4-tetrahydroquinoxaline-6-sulfonamide) and **19** (2-((7-methyl-5,6,7,8-tetrahydrobenzo[4,5]thieno[2,3-d]pyrimidin-4-yl)thio)-N-(m-tolyl)acetamide) showed the best potencies against *Sm*CD1, with IC_50_ values of 83.6 µM and 102.5 µM, respectively (Fig. 5A-B). To escape the affinity-biased selection and optimisation towards larger ligands, we calculated the binding efficiency index defined as BEI = *p*IC_50_/MW (BEI)^54^ ^,^ ^55^ of our most potent hits. Prioritising hits according to their BEI values allows smaller low affinity compounds to be attractive for further hit-to-lead optimisation. Compounds **5** and **19** presented BEI values (12.3 and 10.4, respectively) comparable to positive control pepstatin (BEI = 11.9). However, our hits have lower molecular weights (compound **5** = 331 Da, compound **19** = 384 Da) than pepstatin (685.9 Da), and therefore have greater potential for prospective hit-to-lead optimisation. Since BEI rank compounds on a negative logarithmic scale (pIC_50_), an increase or decrease of one unit implies a corresponding 10-fold change of potency per MW.^54^ ^,^ ^55^

*Rationalising SmCD1 inhibition by compounds 5 and 19* - Understanding the interaction pattern of our most promising hits *i.e.*, compounds **5** and **19**, into the *Sm*CD1 active site is essential for designing more potent analogs.^16^ ^,^ ^53^

**Fig. 6:**
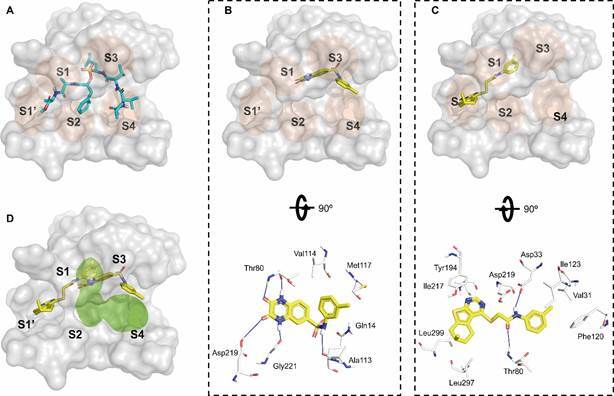
rationalising *Schistosoma mansoni* cathepsin D-like aspartyl protease 1 (*Sm*CD1) inhibition.^16^ (A) phosphonate inhibitor IVA-Val-Val-Leu(P)-(O)Phe-Ala-Ala-Ome bound to human pepsin. (B-C) Binding interactions of *Sm*CD1 with compounds 5 and 19 predicted by docking. (D) Superimposition of docked poses for compounds **5** and **19**, highlighting in green regions for exploring structural optimisation by fusion or growth. The amino acids involved were represented as CPK coloured sticks. The hydrogen bond interactions with protein backbone and side-chain atoms are shown in blue lines.

As shown in Fig. 6A for human pepsin, it is common to find aspartic protease inhibitors that bind to S_1_-S_4_ pockets (*e.g.*, phosphonate inhibitor IVA-Val-Val-Leu(P)-(O)Phe-Ala-Ala-Ome^56^), PDB ID: 1QRP, whereas the pockets S_1ʹ_-S_4ʹ_ remain empty. According to the docking poses, compounds **5** and **19** bind to the pockets S1-S3 and S1’-S1 (see Fig. 6B-C, respectively).

The binding mode of compound **5** in active site of *Sm*CD1 (Fig. 6B) can be generalised as follows: the quinoxaline-2,3-dione moiety can form hydrogen bonds (represented as blue lines) with the carbonyl/amine backbone of the amino acid Thr80, Asp219, and Gly221. Thr80 is part of the “Flap”, a flexible region of the enzyme, which can be in different positions, according to the state of the protein. The possible function of this structure is to facilitate the binding of the substrate or inhibitor to the enzyme,^57^ with a key role in this enzyme-binding interaction.^58^ In addition, the phenyl ring in compound **5** forms van der Waals interactions with pocket formed by Val114 and Met117, whereas nitrogen of sulfonamide group forms a hydrogen bond with Ala113.

On the other hand, compound **19** is predicted to interact through the substrate-binding cleft (Fig. 6C) in a different way, as follows: the amide group can form hydrogen bond interactions with Asp33 and the backbone of Thr80 whereas aromatic and aliphatic rings form van der Waals interactions with Ile123, Val31, Phe120, Leu297, Leu299, Tyr194, and Ile217.

Considering that our hit compounds do not completely occupy the S_1_-S_4_ pockets, we believe that their chemical structures still need to be optimised to generate larger molecular entities with improved potency and BEI values against *Sm*CD1. Since both hits partially occupy the S_1_ pocket, we emphasise the possibility of carrying out a molecular hybridisation through the fusion of quinoxaline-2,3-dione moiety of compound **5** and the phenyl ring of compound **19**. Another relevant possibility for structural optimisation involves the growth of our hits, in order to increase the size and occupation of the active site toward the S_4_, S_2_, and S_1’_ regions (Fig. 6D).

*Selectivity towards parasite proteases* - Compound **5** could not inhibit more than 45% of the *BtCD* activity at the highest concentration evaluated (500 μM due to solubility limitation), indicating it’s IC_50_ value for *BtCD* will be over 500 μM. However, Table shows that while compounds **5** and **19** were the most potent against recombinant *Sm*CD1, these compounds were not selective, being able to inhibit porcine pepsin with similar or even higher potencies. Compound **22** was a potent inhibitor of porcine pepsin but not against *Sm*APs in the AWAE or recombinant *Sm*CD1. On the other hand, compound **50** was the most potent against the AWAE aspartic protease activity (IC_50_ = 77.7 μM) while not showing significant inhibition against pepsin or *Sm*CD1 up to tested concentration 500 μM.

**Fig. 7:**
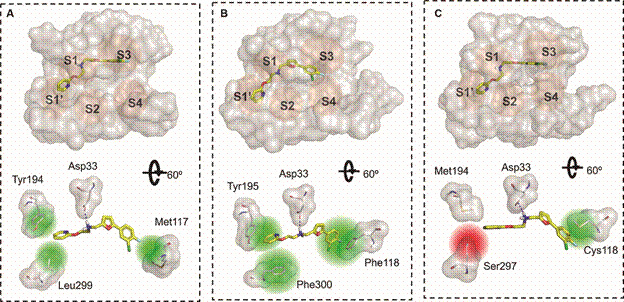
binding modes of compound 50 into the active sites of *Schistosoma mansoni* cathepsin D-like aspartyl proteases (*Sm*CDs). (A) *Sm*CD1, (B) *Sm*CD2, and (C) *Sm*CD3 predicted by docking. Amino acid residues are shown as gray colored sticks. The hydrogen bonding interactions with protein backbone and side-chain atoms are shown in dashed blue lines. Green regions indicate van der Waals or π-stacking interactions. The red sphere indicates a region where steric hindrance may occur.

Although compound **50** has been prioritised as a potential dual inhibitor of *Sm*CD1 and *Sm*CD2, our experimental validation indicates that this compound has inhibitory activity on the AWAE assay but not on *Sm*CD1. Therefore, we hypothesise that the observed inhibition of the AWAE activity by compound 50 is due to inhibition of *Sm*CD2 present in this extract. In view of this, differences in the binding modes of compound **50** at the active sites of the three *Sm*CDs were exploited to rationalise its preference for *Sm*CD2. According to the docking calculations, compound **50** binds to the pockets S1’-S3 of *Sm*CD1 (Fig. 7A) and *Sm*CD2 (Fig. 7B). Although the binding modes are quite similar, the pyridine and aromatic rings of compound **50** make π-stacking interactions with the Tyr195, Phe300 and Phe118 residues of *Sm*CD2 (Fig. 7B). The planar binding profile does not occur in the active site of *Sm*CD1 (Fig. 7A), since the corresponding residues in this region (*i.e.*, Leu299 and Met117) do not have an aromatic character. On the other hand, compound 50 was probably not ranked as a putative hit for *Sm*CD3 because it is sterically hindered by Ser297 in the active site (Fig. 7C).

**Fig. 8:**
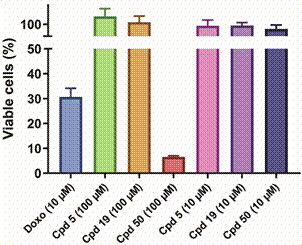
effect on viability of WSS-1 cells after treatment with hit compounds **5**, **19** and **50**, at concentrations of 10 and 100 μM, for 48 hours. Doxo = doxorubicin; Cpd = compound. Bars represent the percentage of viable cells, calculated as the mean ± standard error.

*Cytotoxicity and S. mansoni adult worms ex vivo assay* - In order to evaluate the antischistosomal effect and safety at the cellular level, hit compounds **5**, **19** and **50** were tested on phenotypic assays against human WSS-1 cells and adult *S. mansoni* worms. Compounds **5** and **19** were non-toxic against WSS-1 cells for up to 100 µM. On the other hand, compound **50** was safe only at 10 µM, having decreased cell viability by more than 90% at 100 µM (Fig. 8).

**Fig. 9:**
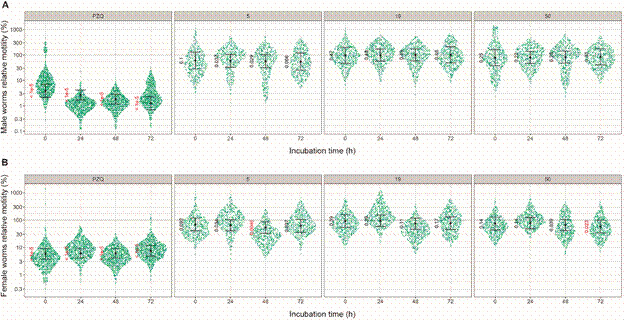
relative motility of male (A) and female (B) adult *Schistosoma mansoni* worms incubated for up to 72 h with 10 µM of compounds **5**, **19**, **50** and praziquantel (PZQ). The x-axis of each graph represents the incubation time (immediately and after 24, 48 and 72 h of compound addition) with the test compounds while the y-axis represents the motility of the treated parasites relative to the control group (0.1 % DMSO) in the logarithmic scale. Each green dot represents single motility measurement of a worm. The estimated effects of compounds at each incubation time and their confidence intervals (95%) are represented by black dots and error bars, respectively. Motility estimates of treated worms whose confidence intervals do not touch the dashed line show statistically significant difference in relation to those of controls. The values shown next to the estimates represent the posteriori probability of the effect of a compound being null or opposite to the estimated effect at that time of incubation. In red, the effects considered statistically different from the effect of the control group and, in black, those that are not statistically significant (cutoff of 0.025). The plots are representative of two independent experiments and were generated with the R ggforce package (https://cran.r-project.org/web/packages/ggforce/index.html).

In antichistosomal *ex vivo* assays, using female and male adult worms, compounds were evaluated at 10 µM for incubation times varying from 0 (just after compound addition) to 72 h incubation. Unfortunately, due to compound solubility limitations, we could only test the compounds at 10 μM concentration, which is the highest possible due to the carried over DMSO concentration (0.1%) from compound stock solutions, above which is toxic to the parasite. The results are shown in Fig. 9.

As shown in Fig. 9, distinctly form the reference drug PZQ, none of the compounds could kill adult worms as judged by lack of substantial decrease in worm motility. Compounds **5** and **50** presented a slight decrease on female worms motility on late incubations times (48 or 72 h). This apparent selective effect is consistent with the higher host protein digestion activity on female worms.^8^ ^,^ ^9^ ^,^ ^10^ It is important to note that, the compounds were evaluated *ex vivo* at a concentration about 10 times lower (10 µM) than their respective IC_50_ values for EAV and/or *Sm*CD1 (Table). At 10 µM, a maximum inhibition of 35% of *Sm*APs activity was expected [Supplementary data (Table II)]. Thus, considering that statistically different effects on motility would result from the inhibition of *Sm*APs, the *ex vivo* results (Fig. 9) are consistent with the results of enzymatic activity.

*In conclusion* - The inhibitory capacity of compounds selected by virtual screening was tested on aspartyl protease activities of the recombinant enzyme *Sm*CD1, aqueous extract of *S. mansoni* adult parasites and the homologous mammalian enzyme, porcine pepsin. Of a total of 20,000 compounds submitted to the virtual screening, the best ranked 50 were selected and tested on such enzymes. Of these, three compounds, **5**, **19** and **50** had the most interesting experimental enzyme inhibition results. Despite the lack of potent anti-parasite effect, under the *ex-vivo* assay condition, the results reported herein allow us to conclude that compounds **5** and **19** are relatively safe new chemical scaffolds that can be optimised into more potent and selective inhibitors of *Sm*CD1 for the development of new antischistosomal drugs.
