# Supplementary material for: Discovery of new Schistosoma mansoni aspartyl protease inhibitors by structure-based virtual screening
Source: Mem Inst Oswaldo Cruz. 2023 Sep 1;118:e230031. doi: 10.1590/0074-02760230031 (PMC10481938; doi:10.1590/0074-02760230031)
Supplement: Supplementary file 1 [file 1678-8060-mioc-118-e230031-s.pdf]

TABLE I

Grid boxes of *Schistosoma mansoni* cathepsin D-like aspartyl protease 1, 2 and 3 (*Sm*CD1, *Sm*CD2, and *Sm*CD3, respectively) used for molecular docking calculations

Model	Coordinates (*x*, *y*, *z*)	Box volume (Å)	Outer shape (Å)
*Sm*CD1	22.2 *vs* 29.2 *vs* 21.3	13.8	4.08
*Sm*CD2	23.3 *vs* 29.7 *vs* 20.8	14.4	3.98
*Sm*CD3	21.0 *vs* 28.5 *vs* 20.2	12.0	3.82

TABLE II

Inhibition of the enzymatic activity of pepsin, adult worm aqueous extract (AWAE) and *Schistosoma mansoni* cathepsin D-like aspartyl protease 1 (*Sm*CD1), observed in the presence of 10 μM of compound. Values represent average of triplicate of single experiment, with coefficient of variation (CV%) indicated in parentheses

	Enzymatic activity inhibition (%)		
Compound	Pepsin	AWAE	*Sm*CD1
Pepstatin	94.9 (22)	98.7 (7)	99.8 (2)
1	5.60 (12)	28.3 (5)	0.40 (5)
2	19.0 (8)	2.90 (1)	.16.2 (12)
3	-20.8 (23)	18.5 (14)	-1.40 (14)
4	-8.1 (15)	5.70 (4)	-0.60 (15)
5	44.2 (23)	23.0 (14)	24.3 (5)
6	0.0 (19)	12.6 (10)	-11.8 (8)
7	14.4 (9)	24.9 (5)	-5.80 (5)
8	1.80 (28)	0.00 (4)	-10.4 (6)
9	-10.6 (7)	5.50 (6)	-20.1 (5)
10	-1.30 (23)	9.90 (2)	10.1 (12)
11	0.50 (15)	10.1 (7)	-11.1 (9)
12	-3.50 (19)	32.0 (11)	-10.5 (15)
13	-4.20 (12)	23.1 (7)	-12.9 (2)
14	-6.90 (6)	24.3 (11)	-18.5 (11)
15	-11.0 (4)	-0.50 (14)	-21.2 (5)
16	-7.10 (4)	7.20 (7)	5.40 (10)
17	21.9 (8)	6.60 (13)	9.50 (5)
18	-14.5 (9)	-18.0 (14)	6.50 (13)
19	42.4 (24)	9.20 (12)	-15.3 (20)
20	16.3 (16)	-13.6 (1)	-7.90 (4)
21	0.30 (9)	15.0 (14)	12.5 (9)
22	25.4 (6)	16.0 (15)	1.80 (4)
23	-4.30 (1)	-11.6 (3)	5.30 (11)
24	-7.30 (14)	-5.50 (11)	12.5 (7)
25	-38.1 (3)	2.90 (5)	7.80 (4)
26	-31.8 (6)	0.60 (3)	-10.6 (6)
27	-2.30 (3)	-14.5 (0)	1.70 (12)
28	3.00 (6)	16.2 (15)	-11.8 (1)
29	-16.2 (10)	-1.20 (18)	10.6 (8)
30	-17.8 (8)	5.20 (11)	-10.1 (5)
31	-32.9 (1)	15.6 (5)	5.10 (14)
32	34.7 (9)	-7.40 (19)	12.2 (9)
33	17.1 (15)	-4.90 (12)	1.30 (5)
34	3.40 (8)	-2.10 (12)	2.50 (8)
35	-2.60 (10)	1.00 (7)	1.90 (9)
36	-7.40 (16)	-8.50 (8)	3.80 (8)
37	-9.80 (6)	6.90 (6)	-0.30 (2)
38	-18.5 (5)	20.6 (38)	-6.20 (2)
39	-5.40 (0)	-31.8 (5)	-3.00 (12)
40	1.30 (19)	20.6 (10)	1.50 (7)
41	-5.80 (7)	23.3 (9)	2.20 (12)
42	21.4 (27)	0.00 (13)	0.00 (5)
43	-11.8 (20)	22.8 (11)	-1.60 (5)
44	-11.1 (13)	2.90 (6)	3.60 (6)
45	0.00 (23)	14.7 (10)	0.00 (23)
46	22.1 (8)	20.2 (13)	-7.90 (8)
47	0.00 (3)	8.50 (1)	0.00 (3)
48	17.5 (22)	13.9 (14)	-4.50 (4)
49	16.9 (11)	20.1 (14)	-0.70 (7)
50	10.5 (2)	34.5 (8)	3.40 (12)

CC BY
